# IP3 receptor orchestrates maladaptive vascular responses in heart failure

**DOI:** 10.1172/JCI152859

**Published:** 2022-02-15

**Authors:** Haikel Dridi, Gaetano Santulli, Jessica Gambardella, Stanislovas S. Jankauskas, Qi Yuan, Jingyi Yang, Steven Reiken, Xujun Wang, Anetta Wronska, Xiaoping Liu, Alain Lacampagne, Andrew R. Marks

**Affiliations:** 1 Department of Physiology and Cellular Biophysics, Clyde and Helen Wu Center for Molecular Cardiology, Department of Medicine, Columbia University Vagelos College of Physicians and Surgeons, New York, New York, USA.; 2 Department of Medicine, Division of Cardiology, Albert Einstein College of Medicine, Wilf Family Cardiovascular Research Institute, Einstein Institute for Aging Research, New York, New York, USA.; 3 Department of Molecular Pharmacology, Einstein-Sinai Diabetes Research Center (ES-DRC), Fleischer Institute for Diabetes and Metabolism (FIDAM), Einstein Institute for Neuroimmunology and Inflammation, Albert Einstein College of Medicine, New York, New York, USA.; 4 International Translational Research and Medical Education (ITME) Consortium, Department of Advanced Biomedical Science, “Federico II” University, Naples, Italy.; 5 PhyMedExp, University of Montpellier, CNRS, INSERM, CHRU Montpellier, Montpellier, France.

**Keywords:** Cardiology, Cell Biology, Calcium channels, Calcium signaling, Cardiovascular disease

## Abstract

Patients with heart failure (HF) have augmented vascular tone, which increases cardiac workload, impairing ventricular output and promoting further myocardial dysfunction. The molecular mechanisms underlying the maladaptive vascular responses observed in HF are not fully understood. Vascular smooth muscle cells (VSMCs) control vasoconstriction via a Ca^2+^-dependent process, in which the type 1 inositol 1,4,5-trisphosphate receptor (IP3R1) on the sarcoplasmic reticulum (SR) plays a major role. To dissect the mechanistic contribution of intracellular Ca^2+^ release to the increased vascular tone observed in HF, we analyzed the remodeling of IP3R1 in aortic tissues from patients with HF and from controls. VSMC IP3R1 channels from patients with HF and HF mice were hyperphosphorylated by both serine and tyrosine kinases. VSMCs isolated from IP3R1^VSMC–/–^ mice exhibited blunted Ca^2+^ responses to angiotensin II (ATII) and norepinephrine compared with control VSMCs. IP3R1^VSMC–/–^ mice displayed significantly reduced responses to ATII, both in vivo and ex vivo. HF IP3R^1VSMC–/–^ mice developed significantly less afterload compared with HF IP3R1^fl/fl^ mice and exhibited significantly attenuated progression toward decompensated HF and reduced interstitial fibrosis. Ca^2+^-dependent phosphorylation of the MLC by MLCK activated VSMC contraction. MLC phosphorylation was markedly increased in VSMCs from patients with HF and HF mice but reduced in VSMCs from HF IP3R1^VSMC–/–^ mice and HF WT mice treated with ML-7. Taken together, our data indicate that VSMC IP3R1 is a major effector of increased vascular tone, which contributes to increased cardiac afterload and decompensation in HF.

## Introduction

Heart failure (HF) is a complex clinical syndrome that remains a leading cause of mortality despite decades of intense efforts to develop novel therapeutics (1, 2). Beginning early in HF, neurohormonal signaling pathways are activated in response to cardiac dysfunction (3, 4). HF is marked by increased vascular tone (afterload) that places additional stress on an already damaged heart, promoting HF progression (5–8). Maladaptive ventricular remodeling, in response to increased afterload, worsens cardiac function, leading to reduced cardiac output, multiorgan failure, and ultimately death (6, 7). Studies dissecting the exact role of intracellular calcium (Ca^2+^) signaling in vascular tone and the subsequent acceleration of cardiac remodeling and HF progression are lacking.

Numerous studies have examined the essential role of Ca^2+^ signaling in regulating blood pressure (reviewed in ref. 9). For example, blocking the voltage-operated Ca^2+^ channels located in the plasma membrane of vascular smooth muscle cells (VSMCs) is an accepted treatment for hypertension (10, 11). Furthermore, the deletion of all inositol 1,4,5-triphosphate receptor (IP3R) isoforms in VSMCs has been shown to significantly reduce hypertension (12–14). However, the role of type 1 IP3R (IP3R1), the main Ca^2+^ channel on the sarcoplasmic reticulum (SR) of VSMCs, as well as its downstream signaling, has yet to be fully elucidated in the context of increased afterload during HF. Of note, in patients with end-stage HF, IP3R1 mRNA and protein levels are upregulated (2, 15, 16), and when cardiac function is reduced, enhanced vascular tone is known to be a prominent response (13, 17).

Since IP3R1 is the major source of SR Ca^2+^ release in VSMCs (12, 18), we sought to determine whether IP3R1 in VSMCs can be linked to VSMC-mediated vasoconstriction and, by extension, to progression to decompensated HF.

## Results

### Biochemical remodeling of VSMC IP3R1 in aortic tissue of patients with HF.

**Figure 1:**
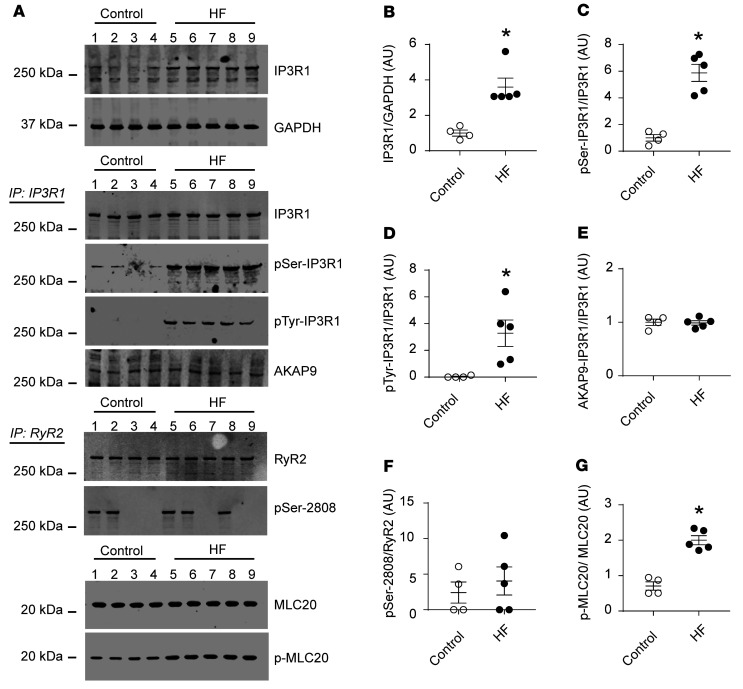
IP3R1 remodeling in aortic tissues from patients with HF. (**A**) Representative immunoblots showing total IP3R1 expression, phosphorylation of serine/tyrosine kinases, and AKAP9 binding to the channels, as assessed by immunoprecipitation (IP: IP3R1); RyR2 phosphorylation levels of PKA-induced RyR2 phosphorylation on serine 2808 (pSer-2808); and p-MLC20 levels in aortic tissues from patients with HF (*n =* 5) and controls (*n =* 4). (**B**–**G**) Quantification of the immunoblots shown in **A**. Individual values with the mean ± SEM are shown. *P < 0.05 versus control, by 2-tailed Student’s *t* test.

To evaluate the role of the VSMC inositol IP3R1 in HF, we obtained aortic tissues from patients with HF and their age-matched controls from the Columbia University Biobank (see Supplemental Table 1; supplemental material available online with this article; https://doi.org/10.1172/JCI152859DS1) and assessed the biochemical remodeling of IP3R1 channels in these tissues. IP3R1 expression and its serine/tyrosine kinase phosphorylation were significantly increased in HF arteries compared with controls (Figure 1, A–D). Using immunoprecipitation, we also found that IP3R1 bound A-kinase–anchoring protein 9 (AKAP9), a scaffold protein known to anchor a pool of protein kinase A (PKA) to the channels, thereby promoting its chronic phosphorylation (Figure 1, A and E, and refs. 19, 20). Interestingly, we noted that phosphorylation of other Ca^2+^ channels such as ryanodine receptor type 2 (RyR2) was unchanged (Figure 1, A and F). A key pathway of Ca^2+^-activated vascular muscle contraction, the MLC20 (21), was hyperphosphorylated in arteries from patients with HF compared with age-matched controls (Figure 1, A and G).

### Generation and characterization of VSMC-specific IP3R1-KO mice.

In order to dissect the molecular mechanisms underlying the increased vascular tone in HF, we examined the role of IP3R1 channels in VSMCs, because Ca^2+^ dynamically controls VSMC contraction (15), and IP3R1 is the major intracellular Ca^2+^ release channel on VSMC SR (12, 18). Therefore, we generated VSMC-specific IP3R1-KO mice (IP3R1^VSMC–/–^). Exon 4 of *Itpr1* was targeted by flanking it with loxP sites as we previously described (22, 23). Mice harboring the IP3R1^fl/fl^ allele were crossed with Sm22α^Cre^-transgenic mice to obtain a VSMC-specific ablation of IP3R1. We did not detect IP3R1 expression in VSMCs isolated from IP3R1^VSMC–/–^ mice compared with IP3R1^fl/fl^ littermates (Supplemental Figure 1, A and B). Immunofluorescence analyses did not detect IP3R1 protein in VSMCs from IP3R1^VSMC–/–^ mice, in either aortas or mesenteric arteries, but as a positive control, IP3R1 protein was detected in endothelial cells in the same sections (Supplemental Figure 1, C and D). Moreover, protein expression (Supplemental Figure 1, A and B) and levels of mRNA encoding the 2 other isoforms of IP3R, IP3R2 and IP3R3, did not exhibit compensatory upregulation in VSMCs from IP3R1^VSMC–/–^ mice (Supplemental Figure 2, A–C).

IP3R1^VSMC–/–^ mice survived to adulthood and had normal renal and cardiac development (Supplemental Figure 2D) and a normal left ventricular ejection fraction (LVEF) at baseline (Supplemental Table 2). Thus, we were able to generate viable mice harboring VSMC-specific deletion of IP3R1 without disturbing the baseline hemodynamics or cardio-renal development.

### Effects of VSMC IP3R1 deficiency on vascular contractility.

**Figure 2:**
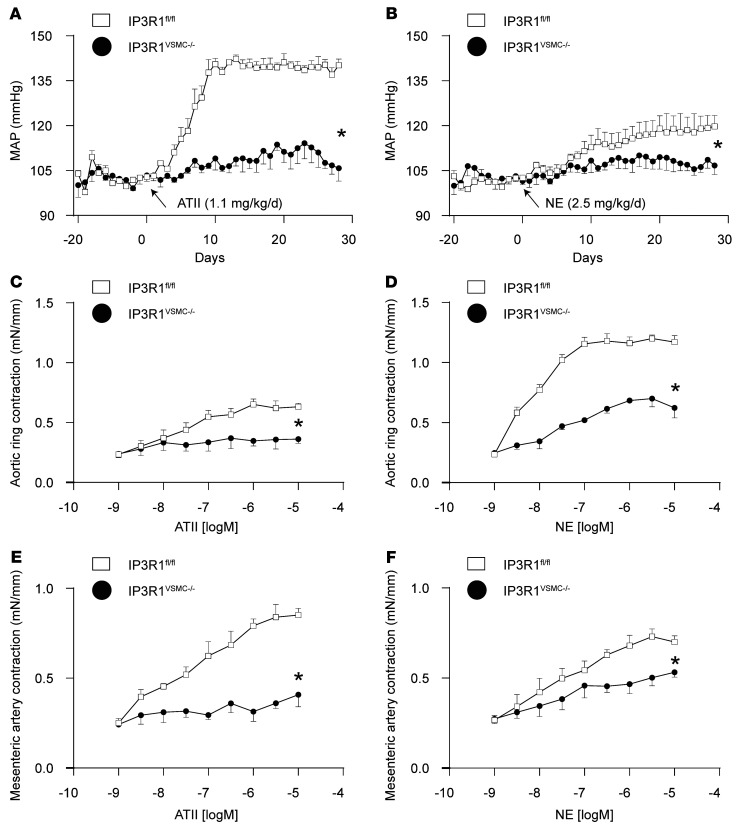
Global evaluation of vascular responses in IP3R1^VSMC–/–^ mice. (**A** and **B**) MAP was measured by radiotelemetry in IP3R1^fl/fl^ and IP3R1^VSMC–/–^ mice at baseline (days –20 to 0) and following administration of ATII (**A**) or NE (**B**) via miniosmotic pumps. *n* ≥6 mice/group. *P < 0.05 versus IP3R1^fl/fl^ mice, by repeated-measures ANOVA. (**C**–**F**) Vascular reactivity of aortic segments (**C** and **D**) and third-order mesenteric arteries (**E** and **F**) in response to ATII (**C** and **E**) and NE (**D** and **F**). Data are presented as the mean ± SEM. mN, millinewton. *n* = 8–20 rings from at least 6 mice per group. *P < 0.05 versus IP3R1^fl/fl^ mice, by repeated-measures ANOVA.

To assess the normal physiological role of IP3R1 in vascular smooth muscle contractility, we examined the effects of vasoconstrictors both in vivo and in vitro in IP3R1^VSMC–/–^ and control IP3R1^fl/fl^ mice. IP3R1^VSMC–/–^ mice displayed significantly blunted responses to angiotensin II (ATII) and norepinephrine (NE) in vivo (Figure 2, A and B). Similarly, aortic ring segments from KO mice exhibited blunted responses to ATII and NE ex vivo (Figure 2, C and D). We extended these results using denuded mesenteric artery preparations (Figure 2, E and F). VSMCs isolated from IP3R1^VSMC–/–^ mice had significantly blunted Ca^2+^ responses to ATII and NE compared with VSMCs isolated from IP3R1^fl/fl^ littermates. IP3R inhibitors (xestospongin C [XSC] or 2-aminoethoxydiphenyl borate [2-APB]) blocked the Ca^2+^ response to ATII and NE in VSMCs isolated from IP3R1^fl/fl^ mice (Supplemental Figure 3, A–E).

### Role of VSMC IP3R1 in vascular responses in ischemic HF.

Impaired cardiac function activates compensatory neurohormonal systems. Initially, within days to weeks following myocardial infarction (MI), the neurohormonal responses to impaired cardiac function can support cardiac output, blood pressure, glomerular filtration rate, and blood flow to essential organs. However, prolonged neurohormonal activation that includes chronic sympathetic stimulation becomes maladaptive and is associated with increased peripheral vascular tone, which puts a mechanical load on an already impaired heart and promotes progressive cardiac dysfunction, ultimately leading to severe HF and decompensation affecting multiple organs (4, 24–26).

**Figure 3:**
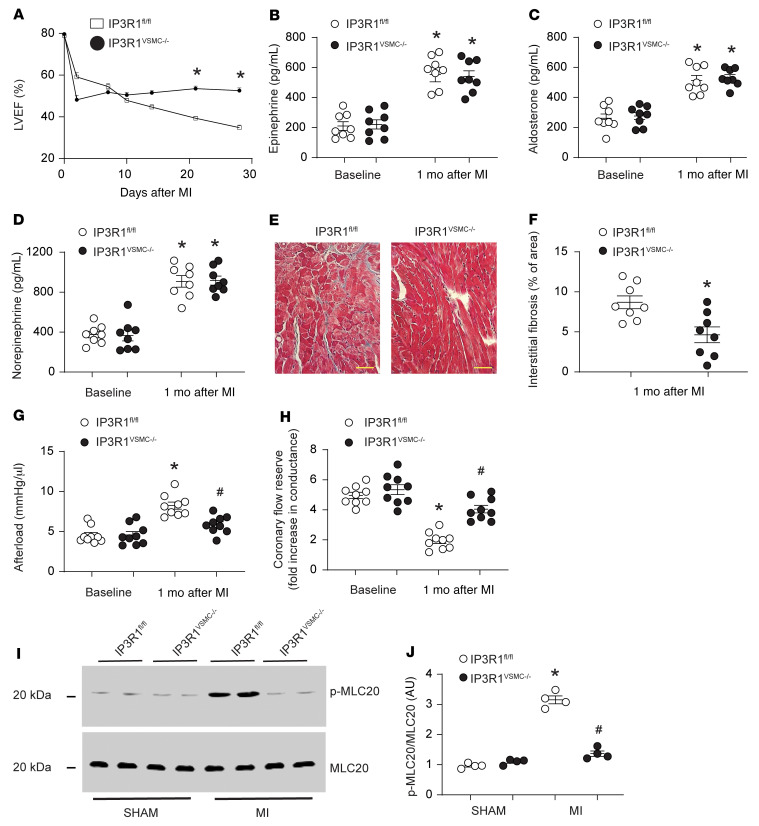
Functional role of VSMC IP3R1 in HF. (**A**) LVEF evaluated by serial echocardiography following surgical ligation of the LAD. (**B**–**D**) Neurohormonal activation in HF assessed by measuring blood levels of catecholamines and aldosterone. (**E**) Representative Masson’s trichrome–stained images showing interstitial cardiac fibrosis. Scale bars: 50 μm. (**F**) Quantification of interstitial cardiac fibrosis. (**G**) Measurement of cardiac afterload (ratio of end-systolic pressure and stroke volume); other hemodynamic parameters are reported in Supplemental Table 2. (**H**) Coronary flow reserve determined in vivo in IP3R1^fl/fl^ and IP3R1^VSMC–/–^ mice. (**I**) Representative immunoblots of denuded mesenteric arteries (*n* ≥8 mice/group) showing MLC protein phosphorylation levels in sham-operated and MI IP3R1^fl/fl^ and IP3R1^VSMC–/–^ mice. (**J**) Quantification of results in **I**. Data are shown as individual values with the mean ± SEM. *P < 0.05 versus sham, by 2-tailed Student’s *t* test; ^#^*P* < 0.05 versus MI IP3R1^fl/fl^ mice, by repeated-measures ANOVA.

To evaluate vascular responses in postischemic HF, we used a murine model of MI achieved by ligation of the left anterior descending (LAD) coronary artery (16). Following MI, we measured cardiac function by cardiac ultrasound. Compared with sham-operated IP3R1^fl/fl^ mice, 4 weeks after surgery, the sham-operated IP3R1^VSMC–/–^ mice had a marked reduction in cardiac function manifested as a decrease in LVEF from approximately 80% to approximately 37% (*P <* 0.05). In contrast, the reduction in cardiac function 4 weeks after MI was significantly attenuated in IP3R1^VSMC–/–^ mice, manifested as a decrease in LVEF from approximately 79% to approximately 47% (*P <* 0.05 compared with post-MI IP3R1^fl/fl^ mice), despite the same degree of myocardial damage measured by infarct size and troponin I levels (Figure 3A and Supplemental Table 2).

MI is associated with a hyperadrenergic state and renin-angiotensin activation that are causally linked to cardiac remodeling, which includes fibrosis and maladaptive cardiac hypertrophy (27–31). We used IP3R1^VSMC–/–^ mice to examine the role of IP3R1 in the VSMC response to neurohormonal activation in HF. Activation of the adrenergic and renin-angiotensin systems was equivalent in IP3R1^fl/fl^ and IP3R1^VSMC–/–^ mice (Figure 3, B–D). However, collagen deposition was markedly attenuated in IP3R1^VSMC–/–^ mice compared with IP3R1^fl/fl^ mice 4 weeks after MI (Figure 3, E and F). Collagen isoforms 1a1, 1a2, 3a1, 8a1, connective tissue growth factor (CTGF), and TGF-β1 mRNAs were significantly reduced in failing hearts from IP3R1^VSMC–/–^ mice compared with that seen in IP3R1^fl/fl^ littermate mice (Supplemental Figure 4). In HF, VSMCs undergo remodeling that includes hyperplasia and increased deposition of extracellular matrix (32). VSMCs isolated from IP3R1^fl/fl^ mice showed reduced expression markers of the nonproliferative state (calponin, smooth muscle myosin heavy chain [SM-MHC]) and augmented levels of markers of the proliferative state (osteopontin, vimentin). In contrast, we observed no significant changes in the expression of these markers in IP3R1^VSMC–/–^ mice (Supplemental Figure 5). Indeed, cardiac afterload was significantly reduced in IP3R1^VSMC–/–^ mice (Figure 3G). Furthermore, when compared with IP3R1^fl/fl^ littermates, IP3R1^VSMC–/–^ mice had significantly higher cardiac contractility (Supplementary Supplemental Table 2) and coronary artery blood flow reserve (Figure 3H).

MLCK is a Ca^2+^-calmodulin–activated (CAM) serine/threonine protein kinase that phosphorylates MLC and activates smooth muscle contraction (21). We sought to determine whether Ca^2+^ release from intracellular stores via IP3R1 activates MLCK. We found that MLC20 phosphorylation was increased in VSMCs from post-MI IP3R1^fl/fl^ mice but not in post-MI IP3R1^VSMC–/–^ mice, strongly suggesting that MLCK Ca^2+^-dependent activation was downstream of IP3R1 (Figure 3, I and J).

### Myogenic tone is decreased in post-MI IP3R1^VSMC–/–^ mice.

**Figure 4:**
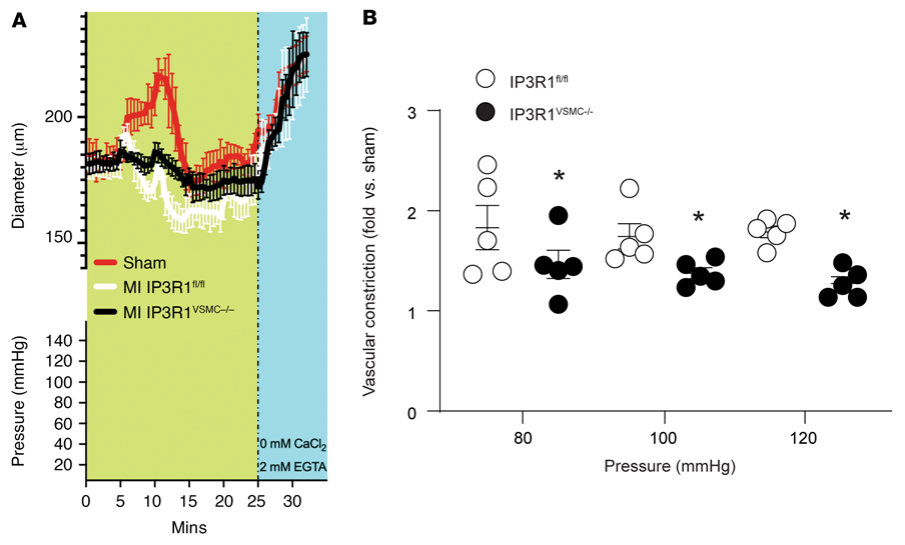
VSMC IP3R1 deletion attenuates peripheral vascular constriction induced by HF. (**A**) Myography traces recorded in cannulated mesenteric arteries during stepwise incremental increases in pressure (namely 80, 100, and 120 mmHg) at a constant temperature of 37°C in regular buffer (green background) and a Ca^2+^-free buffer (light blue background). For clarity, only the sham curve for IP3R1^fl/fl^ mice is shown (not significantly different from the responses in IP3R1^VSMC–/–^ mice). (**B**) Individual myogenic tone values are shown as the mean ± SEM. *n =* 5/group. *P < 0.05 versus IP3R1^fl/fl^ mice, by 2-tailed Student’s *t* test. Of note, the difference in the myogenic contractile response (*P < 0.05) cannot be attributed to modifications in wall thickness (see Supplemental Figure 6).

To confirm the mechanistic role of the VSMC IP3R1 channel in the enhanced vascular tone during HF, we compared the myogenic responses of third-order mesenteric arteries (~150 μm) freshly isolated from IP3R1^fl/fl^ and IP3R1^VSMC–/–^ mice 4 weeks after MI. Diameter changes in pressurized segments of IP3R1^fl/fl^ and IP3R1^VSMC–/–^ mesenteric arteries in the absence of intraluminal flow were measured at 40, 80, and 120 mmHg (Figure 4, A and B). We found that ablation of IP3R1 channels in VSMCs significantly (*P* < 0.05) attenuated the increased myogenic tone observed in HF. This increase in myogenic tone was not caused by vessel remodeling, as the arterial wall thickness was unchanged at a range of pressure from 20 to 120 mmHg (Supplemental Figure 6).

### MLCK is downstream of IP3R1.

To determine whether the effects of IP3R1 deficiency in post-MI VSMCs could be mediated by MLCK, we treated WT MI mice with the MLCK inhibitor ML-7 (33, 34) (1 mg/kg/d for 4 weeks after MI). ML-7–treated WT MI mice exhibited significantly attenuated progression toward decompensated HF (Supplemental Figure 7A and Supplemental Table 3). Activation of the adrenergic and renin-angiotensin systems was equivalent in WT MI placebo–treated mice and WT MI mice treated with ML-7 (Supplemental Figure 7, B–D). Fibrosis was markedly attenuated in ML-7–treated WT MI mice compared with placebo-treated mice (Supplemental Figure 7, E and F). Moreover, cardiac afterload was significantly reduced in ML-7–treated WT MI mice compared with placebo-treated WT MI mice (Supplemental Figure 7G). Furthermore, ML-7–treated WT MI mice showed a significant improvement in myocardial blood flow reserve (Supplemental Figure 7H). MLC20 phosphorylation was blunted in ML-7–treated WT MI mice compared with the untreated mice, suggesting a role for MLCK-mediated transduction of the IP3R1 Ca^2+^ signal in terms of peripheral vasoconstriction via activation of CAM, as previously reported (ref. 35 and Supplemental Figure 7, I and J).

### Role of adrenergic signaling in peripheral vasoconstriction during HF.

**Figure 5:**
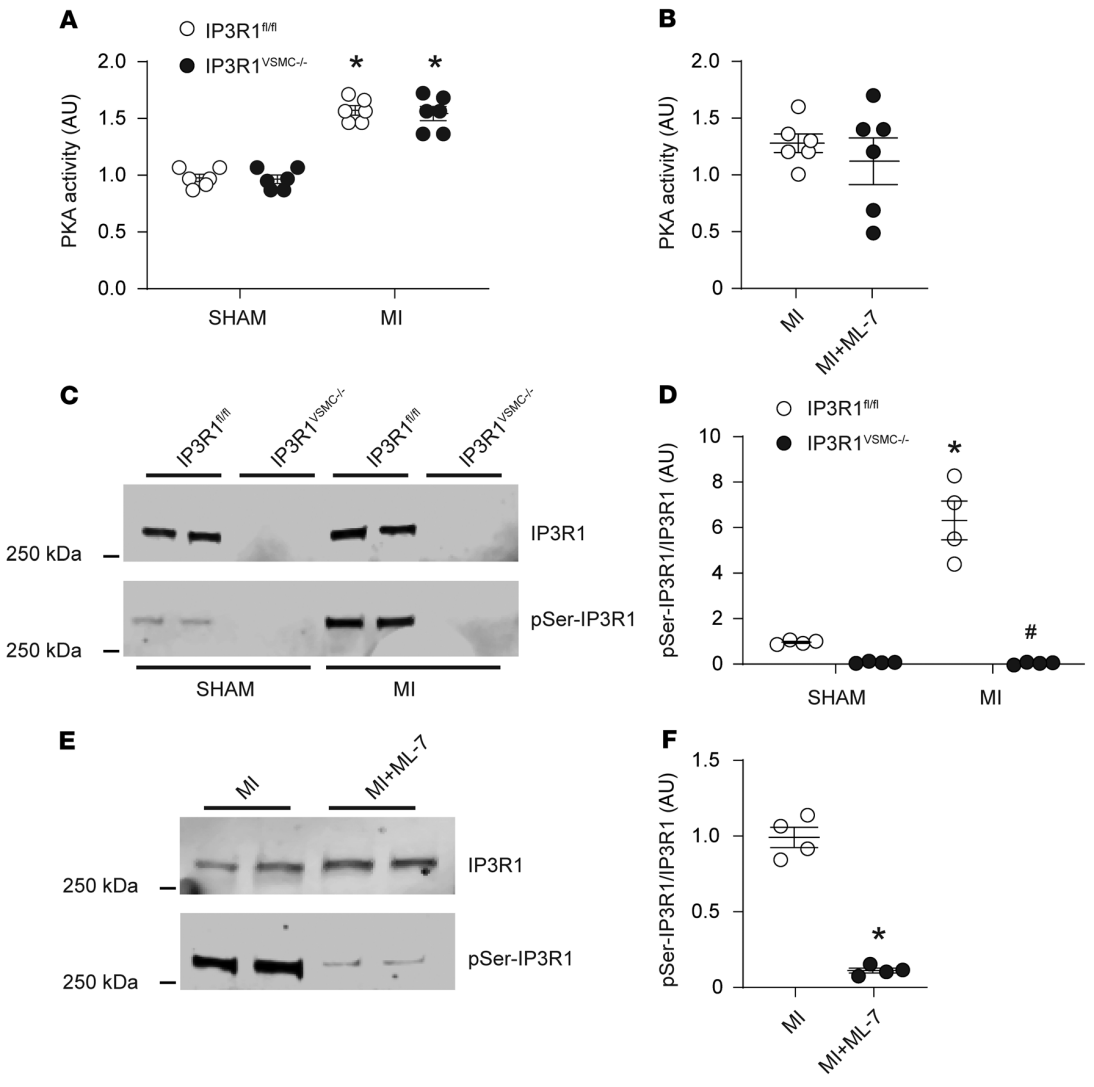
Increased IP3R1 phosphorylation by PKA in VSMCs during HF. (**A**) PKA activity in VSMCs from sham-operated and MI IP3R1^fl/fl^ and IP3R1^VSMC–/–^ mice. (**B**) PKA activity in VSMCs from MI and ML-7–treated MI mice (*n* = 6 mice per group). (**C** and **D**) Representative immunoblots (**C**) and quantification (**D**) showing increased IP3R1 phosphorylation by PKA in VSMCs from IP3R1^fl/fl^ mice after MI compared with sham-operated mice (*n* = 6 mice per group). (**E** and **F**) Representative immunoblots (**E**) and quantification (**F**) showing reduced IP3R1 phosphorylation by PKA in VSMCs from HF-treated mice compared with untreated mice (*n* = 4 mice per group). Individual values are shown with the mean ± SEM. *P < 0.05 for IP3R1^fl/fl^ sham versus IP3R1^fl/fl^ MI mice, IP3R1^VSMC–/–^ sham versus IP3R1^VSMC–/–^ MI mice, and MI mice versus ML-7–treated MI mice; 2-tailed Student’s *t* test. ^#^*P <* 0.05, for IP3R1^fl/fl^ MI versus IP3R1^VSMC–/–^ MI mice; repeated-measures ANOVA.

Chronic catecholamine (e.g., epinephrine and NE) spillover is a hallmark of HF and a strong predictor of mortality in patients with failing hearts (4, 28, 36). Catecholamines bind to β-adrenergic receptors in VSMCs and activate adenylyl cyclase (AC), which produces cAMP, resulting in downstream activation of PKA (36, 37). Active PKA phosphorylates several cellular targets including IP3R1 and RyR2 channels, thereby enhancing their activity (24, 38, 39). We observed that PKA activity was increased in VSMCs from post-MI IP3R1^fl/fl^ and IP3R1^VSMC–/–^ mice compared with sham-operated mice, which is consistent with the increased catecholamine levels detected in these mice (Figure 5A). PKA activity was similar between untreated WT MI and ML-7–treated mice (Figure 5B). IP3R1 channels in VSMCs from post-MI IP3R1^fl/fl^ and WT MI untreated mice were phosphorylated by PKA, whereas such phosphorylation was markedly blunted in ML-7–treated WT HF mice (Figure 5, C–F). Therefore, IP3R1 PKA phosphorylation and activation of the IP3 signaling pathway likely contribute to increased SR Ca^2+^ release and vascular tone in VSMCs during HF.

We also evaluated RyR2 phosphorylation in VSMCs. RyR2 channels are another type of intracellular Ca^2+^ release channel in VSMCs and a target for PKA (38). In VSMCs, RyR2 channels are involved in vasorelaxation. RyR2 clusters in VSMCs are functionally coupled with multiple large-conductance, Ca^2+^-sensitive potassium channels (BK): RyR2 Ca^2+^ release generates a large transient outward K^+^ current that hyperpolarizes the plasma membrane, deactivating voltage-dependent Ca^2+^ influx to cause VSMC relaxation and vasodilation (40–44). We observed an increase in PKA-induced RyR2 phosphorylation in VSMCs of post-MI IP3R1^fl/fl^ mice but not in those of post-MI IP3R1^VSMC–/–^ mice (Supplemental Figure 8, A and B). Furthermore, the phosphoinositol pathway that generates IP3 is known to be coupled to the activation of the cell surface receptors, either G proteins or nonreceptor protein tyrosine kinases. IP3R1 receptors are phosphorylated by tyrosine kinases that modulate the channel activity. We observed increased tyrosine kinase phosphorylation of IP3R1 in post-MI IP3R1^fl/fl^ mice, an event that may further potentiate SR Ca^2+^ release and promote vasoconstriction (Supplemental Figure 8, C and D).

## Discussion

Reducing cardiac afterload is an important therapeutic approach in patients with HF (1, 5, 45, 46). However, most of the vasodilators currently available in clinical practice either increase cardiac output at the expense of increased heart rate, arrhythmias, and mortality, or have negative inotropic effects that limit their use (45–48). Moreover, for the most part, current therapies for HF do not target molecular mechanisms responsible for the elevated vascular tone observed in HF (1, 49). In the present study, we show that IP3R1 channels in VSMCs play a critical role in the cardiac response to neurohormonal activation during HF that consists of fibrosis and hyperplasia presumably due, at least in part, to increased afterload in which IP3R1 is instrumental. This finding is in accordance with previous studies showing that VSMCs undergo pathological remodeling that includes hyperplasia and increased deposition of extracellular matrix (32). Interestingly, in our study, markers of proliferation, collagen deposition, and fibrosis were significantly (*P* < 0.05) decreased in IP3R1^VSMC–/–^ and ML-7–treated post-MI mice.

Cardiac afterload, defined as the ratio of end-systolic pressure to stroke volume, reflects arterial vascular resistance, which imposes a functional load on the heart (50). In HF, vasoconstriction of mesenteric arteries becomes excessive in order to maintain normal blood pressure; however, such vasoconstriction increases cardiac workload and reduces myocardial perfusion, which is detrimental for cardiac remodeling. In our models, constriction of resistance arteries and cardiac afterload were both significantly (*P* < 0.05) increased in HF mice, but attenuated in IP3R1^VSMC–/–^ post-MI mice, highlighting the crucial role of VSMC IP3R1 channels in the progression of HF.

Our results are in agreement with the reduced aortic contractile responses to several drugs, including endothelin 1, phenylephrine, serotonin, and the vasopressin mimic U46619, reported in aortas in which the 3 isoforms of the IP3R had been ablated (12). Thus, the IP3R1 channel plays a major role in the contractile response to physiological vasoconstrictors including catecholamines, which are known to be elevated in HF (26, 51, 52). Although the IP3Rs (IP3R1, 2, and 3) have been previously reported to modulate Ca^2+^ release and regulate vascular contractility in hypertension (12), to our knowledge, this is the first study that dissects the role of the VSMC IP3R1 and its direct link to the neurohormonal dysregulation in HF using human aortic tissues and IP3R1^VSMC–/–^ mice. Interestingly, we did not observe any significant change in the expression of other IP3R isoforms (IP3R2 and -3) in our IP3R1^VSMC–/–^ model as a compensatory mechanism (both at the protein and mRNA levels). These results are discordant with those of Lin et al. (12), who reported unchanged IP3R2 proteins but increased IP3R3 isoform expression as a compensation for IP3R1 deletion. This discrepancy could be due to a contamination of their VSMCs by other types of cells (such as endothelial cells) during the isolation of VSMC layers. Moreover, our higher number of backcrossings likely helped stabilize our mouse model and reduce possible compensatory mechanisms.

In addition to the IP3R1 pathway activation that we report in the present study, alterations of VSMC excitability are involved in vasoconstriction during HF. Strong hyperpolarizing currents are required to prevent excessive VSMC depolarization and contractility that cause vasoconstriction. The BK potassium channel is an important contributor to the hyperpolarizing currents in VSMCs (53). HF has been associated with a downregulation of these channels, which promotes vasoconstriction, most likely working in synergy with the elevated cytosolic [Ca^2+^] mediated via IP3R1 channels. Supporting this view, the pressure-sensing signaling pathway through phospholipase C (PLC) and the transient receptor potential channels (TRPC6 and TRPM4) have been reported to mediate contraction of cerebral VSMCs via an IP3R Ca^2+^-dependent mechanism (54, 55), and the interaction between the IP3R and transient receptor potential (TRP) channels seems to be functionally involved in the modulation of vascular tone in cerebral arteries (56). Although these studies did not investigate HF or the specific role of the IP3R1 isoform, they provide strong support for a major role of IP3R1 channels in the regulation of VSMC contractility by Ca^2+^.

In VSMCs, MLCK is a CAM-activated serine/threonine protein kinase that phosphorylates MLC20 and activates VSMC contraction (21). Ca^2+^ release from intracellular stores via the IP3R1 activates MLCK and enhances vasoconstriction. For instance, mechanical stimuli (e.g., pressure <60 mmHg) have been shown to augment SR Ca^2+^ waves in arterial smooth muscle, hence activating MLC20 and increasing vascular tone (57). We found an increase of MLC20 phosphorylation in VSMCs from post-MI IP3R1^fl/fl^ mice but not in post-MI IP3R1^VSMC–/–^ mice, strongly suggesting that MLCK Ca^2+^-dependent activation was downstream of the IP3R1.

Furthermore, WT MI mice treated with the MLCK inhibitor ML-7 exhibited significantly (*P* < 0.05) attenuated fibrosis and cardiac afterload, thus slowing the progression toward decompensated HF. These findings are consistent with previous studies reporting that ML-7 treatment protects the heart against ischemia/reperfusion injury (58). Finally, it is noteworthy to mention that the ML-7 inhibitor targets the ATP binding site of MLCK, which is highly homologous with other kinases, including PKA and PKC (59). Although the compound has been reported to be toxic in vitro at relatively high concentrations (>30 μM; ref. 60), in the present study, we did not see any significant toxicity or off-target action of the drug; in fact, treated mice survived until the end of the experiments and had improved cardiac function. Moreover, PKA activity in ML-7–treated mice was not significantly changed compared with untreated mice.

We found increased PKA activity in VSMCs from post-MI mice, which is consistent with increased catecholamine levels. IP3R1 phosphorylation by PKA was augmented as well. IP3R1 PKA-induced phosphorylation of IP3R1 and activation of the IP3 signaling pathway likely partake in the increased SR Ca^2+^ release and vascular tone in VSMCs during HF. Phosphorylation of IP3R1 was increased in post-MI IP3R1^fl/fl^ and WT MI mice. Similarly, increased serine and tyrosine phosphorylation of IP3R1 was detected in aortas from patients with HF. This finding is in accordance with our previous assays showing that tyrosine phosphorylation of IP3R1 modulates the channel activity and regulates intracellular Ca^2+^ levels (61).

In HF, PKA phosphorylation of IP3R1 channels seems to be a major pathway involved in vasoconstriction, since it is directly linked to increased levels of catecholamines, whereas tyrosine kinase phosphorylation would shift the Ca^2+^ dependence of inactivation of IP3R1 to higher values of cytoplasmic [Ca^2+^] ([Ca^2+^]_cyt_). Our results are consistent with recent reports from other investigators demonstrating the fundamental role of PKA in the regulation of VSMC activity (41, 62, 63).

We also observed PKA phosphorylation of RyR2 channels in mouse VSMCs, which is known to cause VSMC relaxation and vasodilation under normal conditions as opposed to the role of IP3R1 channels (41, 44). Although such phosphorylation is expected to attenuate vasoconstriction, most likely as a compensatory mechanism to promote vasodilation, this was not the case in the current context. This finding might be explained by a decreased expression of the BK channel during HF (64), which would reduce hyperpolarization of the cell membrane, thereby impairing VSMC relaxation. Moreover, in end-stage HF, RyR2 mRNA and protein levels are downregulated, whereas IP3R1 expression is upregulated (2, 15, 16), as also observed in the present study (increased IP3R1 levels in aortic tissues of patients with HF). Finally, in accordance with our study, GWAS data from European ancestry cohorts have shown that blood pressure is associated with the IP3R1 gene (*Itpr1*), corroborating the role of IP3R1 channels in vascular function (65). Further studies are needed to develop specific IP3R1 inhibitors to directly target the channel in the context of HF.

In the present study, we used human aortic tissues from patients with HF and control individuals to investigate the expression and biochemical modifications of IP3R1 channels. These tissues are not necessarily representative of the resistance vessels, which have thicker muscular walls and narrower lumens (66). However, in our mouse model (IP3R1^VSMC–/–^), we observed similar responses to IP3R1 agonists (ATII and NE) in both aortic rings and mesenteric arteries, thus suggesting that the mechanism by which Ca^2+^ regulates IP3R1 activity is very similar in both types of arteries. We did not evaluate the correlation between the loss of IP3R1 in VSMCs and the prevalence of SM22α. However, a region of the SM22α promoter containing 445 base pairs of the 5′-flanking sequence was found to be sufficient to direct the specific expression of a lacZ transgene in mouse embryos in vascular smooth, cardiac, and skeletal muscle lineages in a temporospatial pattern similar to that of the endogenous SM22α gene (67). Finally, RyR2 phosphorylation by PKA in aortic tissues from patients with HF was unchanged and seemed to be more related to the patient’s age. This finding needs to be further investigated in the future with a larger sample size.

In summary, we observed increased expression and phosphorylation of IP3R1 channels in aortic tissues from patients with HF compared with control aortic tissues. We also demonstrate that specific deletion of the IP3R1 in VSMCs attenuated the maladaptive vascular responses commonly observed in HF and slowed the progression toward decompensated HF; this effect was mediated by the phosphorylation of MLC20, which is involved in VSMC contractility.

**Figure 6:**
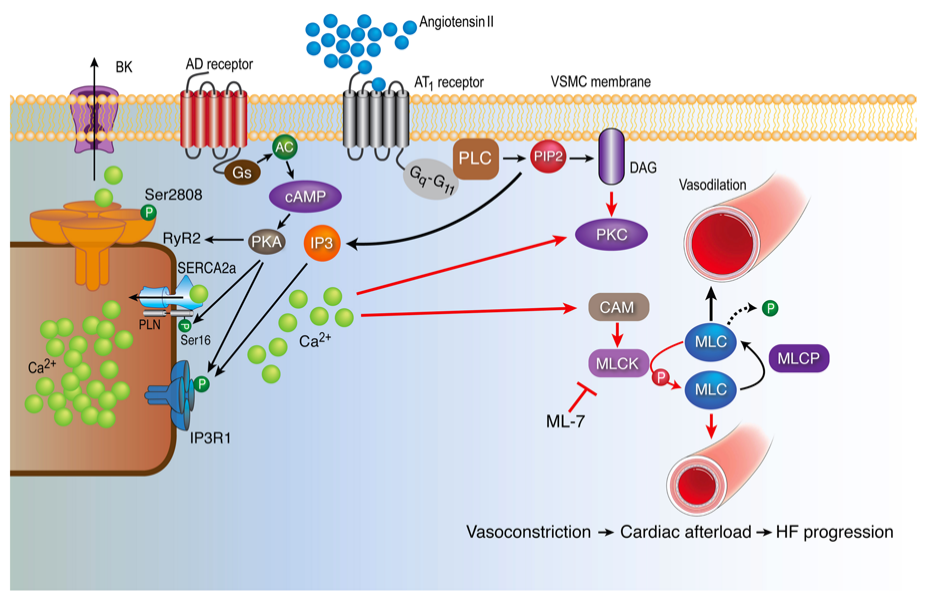
IP3R1 in vascular smooth muscle mediates increased vascular tone during HF. ATII binds to GPCRs (G_q_–G_11_) and activates PLC, which hydrolyzes phosphatidylinositol 4,5-bisphosphate (PIP2), resulting in 2 second messengers: IP3 and diacylglycerol (DAG). IP3 binds to its receptor IP3R1 on the SR, causing Ca^2+^ release into the cytosol. Furthermore, increased catecholamines during HF bind to adrenergic (AD) receptors and activate the Gs protein and AC, leading to increased levels of cAMP. cAMP activates PKA, which phosphorylate the IP3R1 channels, causing further Ca^2+^ release into the cytosol. IP3R1 binds AKAP9, which anchors a pool of PKA to the channel. Of note, PKA phosphorylates RyR2 and SR/ER Ca^2+^-ATPase type 2 (SERCA2a), which play a role in SR Ca^2+^ release and uptake processes, respectively. An increase in the cytosolic Ca^2+^ concentration activates the CAM protein. CAM activates MLCK, which in turn phosphorylates MLC20, leading to smooth muscle contraction and vasoconstriction. Chronic vasoconstriction increases cardiac afterload, thereby promoting decompensated HF. Both genetic depletion of VSMC IP3R1 and pharmacologic inhibition of MLCK with ML-7 attenuate MLCK activation and phosphorylation of MLC20, thus reducing vasoconstriction and cardiac afterload in failing hearts. MLCP, myosin light chain phosphatase; PLN, phospholamban.

Taken together, the data in the present study suggest that VSMC IP3R1 plays an important role in the peripheral vasoconstriction observed in HF (summarized in Figure 6), which contributes to increased afterload and cardiac decompensation during HF progression.

## Methods

### Human studies.

Deidentified human aortic tissues from age-matched patients with HF (*n =* 5) and control individuals (*n =* 4) were obtained from the Columbia University Biobank under the IRB protocol AAAT5397. Characteristics of the patients and controls are shown in Supplemental Table 1.

### Animal studies.

Exon 4 of *Itpr1* was targeted by flanking it with loxP sites (22, 23). The generation of embryonic stem cell–derived embryos has been described elsewhere (16). Mice harboring the IP3R1^fl/fl^ allele (IP3R1^fl/fl^) were bred with Sm22α^Cre^-transgenic mice (The Jackson Laboratory) to obtain mice with a VSMC-specific ablation of IP3R1 (IP3R1^VSMC–/–^). All mice were backcrossed onto a C57BL/6 background for more than 12 generations. All in vivo and in vitro experiments were conducted by operators who were blinded to the genotypes of the animals.

### In vivo experiments.

Transthoracic echocardiography was performed using a 12 MHz probe (Vevo 2100, Visualsonics; ref. 68). Following baseline echocardiography, MI was induced in 5- to 6-month-old mice (16). We choose the MI model among other HF models in order to achieve consistently larger infarct sizes and more uniform HF. A small thoracotomy was performed via the fourth intercostal space, and the lungs were gently retracted to expose the heart. The LAD was located and permanently ligated near its origin between the pulmonary outflow tract and the edge of the left atrium (69, 70). A group of age-matched littermate mice receiving sham ligation underwent the same surgical procedure but without tightening the suture around the artery. Serum concentrations of troponin I were measured 1 day after coronary artery ligation using a commercially available immunoassay kit (16, 70). Myocardial infarct size was expressed as a percentage of the total LV area determined by echocardiography (71). To evaluate cardiac function, echocardiography was performed at regular intervals over the ensuing 4 weeks.

Blood pressure was recorded in conscious, freely moving mice using radiotelemetric transmitters (TA11PA-C10, Data Sciences International) implanted into the aortic arch (16, 68). Data were acquired for 2 minutes every 15 minutes, and the average values for the mean arterial pressure (MAP) were calculated for every time point. Cardiac hemodynamics were assessed in anesthetized mice using a 1.0 F Mikro-Tip catheter (PVR1045, Millar Instruments) connected to a transducer (Gould Instruments Systems), as we previously described and validated (16, 23, 68). In some experiments, mice were treated with ATII (1.1 mg/kg/d) or NE (2.5 mg/kg/d) via miniosmotic pumps (Alzet) implanted subcutaneously, as we previously described (72). Coronary blood flow was determined using 15 μm dyed microspheres of different colors (Triton), processing cardiac tissue and blood samples according to the manufacturer’s instructions. To calculate coronary flow reserve, myocardial blood flow measurements were obtained at basal and after maximal vasodilation (dipyridamole i.v., 5 mg/kg/min for 6 minutes). Blood levels of catecholamines and aldosterone were determined using commercially available ELISA kits (Biomatik), according to the manufacturer’s instructions.

### MLCK inhibitor treatment.

ML-7 hydrochloride (475880, Merck; ref. 73) was administrated to mice via i.p. injection at 1 mg/kg/d for 4 weeks after MI; 0.9% NaCl was used as a vehicle. The untreated mice received the same volume of saline.

### Vascular reactivity and myogenic tone.

Mice were euthanized, and the mesenteric artery and thoracic aorta were rapidly harvested and dissected. Vascular segments were mounted in a Multi Myograph System (Danish MyoTechnology) in a Krebs buffer (119 mM NaCl, 4.7 mM KCl, 2.5 mM CaCl_2_, 1 mM MgCl_2_, 25 mM NaHCO_3_, 1.2 mM KH_2_PO_4_, and 11 mM d-glucose, pH 7.4). Curves for cumulative concentration responses to NE and ATII were determined (68, 74, 75). Vascular myogenic tone was assessed in third-order mesenteric arteries mounted onto 2 glass cannulas in a dedicated pressure myography chamber (Danish MyoTechnology), and diameter changes in response to different pressures were measured (76), according to the manufacturer’s instructions. Arterial segments were first equilibrated at 50 mmHg for 15 minutes in Krebs buffer and then subjected to stepwise increases and decreases (5 minutes each) in intraluminal pressure (80, 100, 120, 100, 80 mmHg); the experiment was concluded by incubating the segments in the same Krebs buffer (except for the absence of CaCl_2_ and the addition of 2 mM EGTA, in order to prevent contraction), repeating the measurements at stepwise increasing pressurizations (80, 100, and 120 mmHg). Myogenic tone was calculated using the values of internal diameters measured during the stepdown pressurizations, as follows: (diameter in the Ca^2+^-free buffer – diameter in the regular buffer)/(diameter in the Ca^2+^-free buffer) × 100. Mesenteric arteries were consistently isolated from the jejunal arcades, where the arteriae rectae are longer. To obtain endothelial denudation (confirmed by a blunted response to acetylcholine), the tip of a hair shaft (moose mane) was used. Throughout the experiments, the temperature was kept constant at 37°C.

### Ca^2+^ imaging.

The mesenteric arteries were rapidly removed and dissected in ice-cold Krebs buffer. The artery was cut into approximately 4 mm^2^ pieces and glued to the glass bottom of a 35 mm cell culture dish (MatTek). The arteries were then loaded with 10 μM Fluo-4 AM (Thermo Fisher Scientific) for 45 minutes, washed 3 times, and then maintained in the following solution: 125 mM NaCl, 4.75 mM KCl, 1.2 mM MgSO_4_, 1.2 mM KH_2_PO_4_, 30 mM HEPES, 10 mM d-glucose, 50 mM taurine, 2 mM CaCl_2_ (pH = 7.4). Confocal imaging was performed by excitation with a 488 nm light from the argon laser of a Zeiss 5 live inverted confocal microscope (40× oil immersion lens) (16). Data were analyzed using ImageJ software (NIH).

### Histology.

Tissues were fixed in 10% formaldehyde overnight and processed for paraffin embedment. H&E staining was performed on 7 μm sections following standard protocols (77). Fibrosis was evaluated by Masson’s trichrome staining (70). For immunofluorescence, paraffin-embedded tissues were deparaffinized and hydrated, and antigens were retrieved before immunostaining with IP3R1 antibody (1:100). Images were captured using a Zeiss 5 live inverted confocal microscope (63× oil immersion lens; ref. 74).

### Real-time reverse transcription quantitative PCR.

Total RNA was extracted using TRIzol (Thermo Fisher Scientific), and cDNA was synthesized using a Thermo-Script RT-PCR System (78, 79). SYBR Green Analysis (Thermo Fisher Scientific) was used to quantify gene expression. All reactions were run in triplicate using the ABI 7500 Fast Real-Time PCR Detection System (16, 78). Reverse transcription quantitative PCR (RT-qPCR) data were analyzed using the comparative CT method and normalized to the expression of β-actin. Primer sequences for gene analysis are listed in Supplemental Table 4.

### Immunoprecipitation and immunoblot analysis.

Tissues were homogenized in 150 mM NaCl, 25 mM Tris-HCl, pH 7.5, 5 mM EDTA, 1% NP-40, 0.4% deoxycholic acid, 1 mM Na_3_VO_4_, and complete protease inhibitors. Protein concentrations were determined using the Bradford assay. Protein (10–20 μg) was size-fractionated on SDS-PAGE gels and immunoblotted. Immunoblotting was performed as previously described and validated (79, 80) using the following antibodies: anti-IP3R1 (sc-271197, Santa Cruz Biotechnology); anti-IP3R2 (sc-398434, Santa Cruz Biotechnology); anti-IP3R3 (610312 , BD Biosciences); anti–phosphorylated MLC20 (anti–p-MLC20) (ab2480, Abcam), anti-MLC20 (ab137063, Abcam); anti-RyR2 (ab55999, Abcam); anti–p-RyR2 (ab59225, Abcam); and anti-GAPDH (ab8245, Abcam).

IP3R1 was immunoprecipitated from 250 μg homogenate using an anti-IP3R1 antibody (sc-271197, Santa Cruz Biotechnology) in 0.5 mL of a modified RIPA buffer (50 mM Tris-HCL, pH 7.4, 0.9 NaCl, 5.0 mM NaF, 1.0 mM Na_3_VO_4_, 1% Triton X-100 and protease inhibitors) for 1 hour at 4°C. The immune complexes were incubated with protein A sepharose beads (Amersham Pharmacia) at 4°C for 1 hour, and the beds were washed 3 times with buffer. Proteins were separated on SDS/PAGE gels (6%) and transferred onto nitrocellulose membranes for 2 hours at 200 mA. Expression of total IP3R1 and p-IP3R1 was probed using the following antibodies: anti-IP3R1 (sc-271197, Santa Cruz Biotechnology); anti–p-IP3R1 (p-serine, Abcam); and anti–p-tyrosine (produced in-house; ref. 61). All immunoblots were developed with the Odyssey system (LI-COR Biosciences), using infrared-labeled anti–mouse and anti–rabbit IgG (1:10,000 dilution) secondary antibodies (79). The intensity of the bands was quantified using LI-COR Image Studio Software (LI-COR Biotechnology).

### PKA activity assay.

Samples were thawed on ice, and PKA activity was determined using a colorimetric assay (139435, Abcam) according to the manufacturer’s instructions. Mesenteric artery lysates containing PKA were added to the plate’s wells in the presence of the supplied ATP, to phosphorylate the immobilized PKA substrate; a specific antibody for the p-PKA substrate binds to the modified immobilized substrate. A secondary antibody labeled with peroxidase was then added to the plate to bind the primary antibody. After incubation and washout, the substrate was added and the absorbance at 450–650 nm was measured using a microplate reader.

### Statistics.

All results are presented as the mean ± SEM. Statistical analyses were performed using an unpaired, 2-tailed Student’s *t* test (for 2 groups) and 1-way ANOVA with a Tukey-Kramer test (for 3 or more groups), unless otherwise indicated. *P* values of less than 0.05 were considered significant.

### Study approval.

All studies were approved by the IACUC of Columbia University (New York, New York, USA) and were conducted according to NIH guidelines (approval no. AC-AAAW5453). The deidentified human specimens were exempted from ethics approval (exemption no. AAAT5397). The methods used in this study adhered to the NIH’s *Guide for the Care and Use of Laboratory Animals* (National Academies Press, 2011). All experiments were performed by operators who were blinded to the genotypes of the animals.

## Author contributions

HD and GS designed the study, performed experiments, analyzed the data, and wrote and edited the manuscript. JG, SSJ, QY, JY, SR, XW, AW, and XL performed experiments and analyzed the data. AL and ARM designed the study, analyzed the data, and wrote and edited the manuscript.

## Supplementary Material

Supplemental data

## Version 1 02/15/2022

Electronic publication
