# Supplementary material for: IP3 receptor orchestrates maladaptive vascular responses in heart failure
Source: J Clin Invest. 2022 Feb 15;132(4):e152859. doi: 10.1172/JCI152859 (PMC8843748; doi:10.1172/JCI152859)
Supplement: Supplemental data [file jci-132-152859-s055.pdf]

Supplementary data

IP3 Receptor Orchestrates Maladaptive Vascular Responses in Heart Failure

Dridi H, Santulli G, et al.

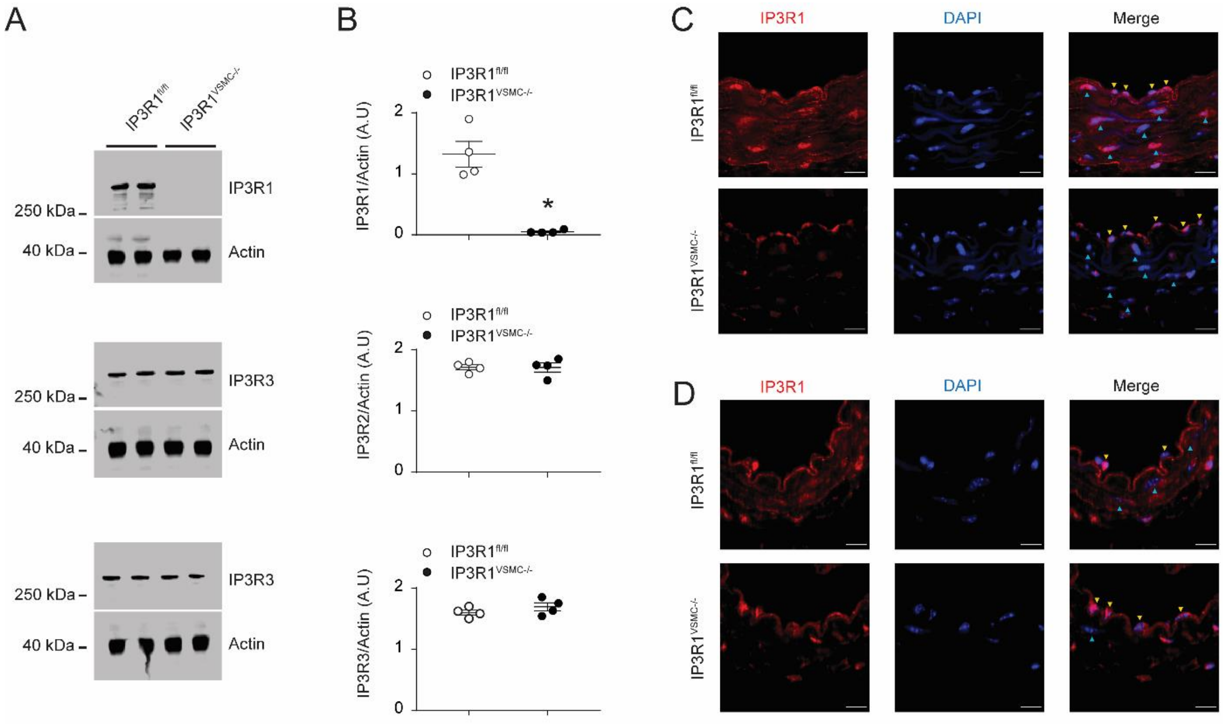

Figure S1. Generation and characterization of IP3R1$^{VSMC-/-}$ mice.

A) Representative immunoblot assessing the expression of IP3R1, IP3R2 and IP3R3 isoforms in denuded aortic segments from IP3R1$^{fl/fl}$ and IP3R1$^{VSMC-/-}$ mice. **B**) Quantification of immunoblots showed in panel **A**. Representative immunofluorescence images of aorta **(C)** and mesenteric arteries **(D)** from IP3R1$^{fl/fl}$ and IP3R1$^{VSMC-/-}$ mice stained for IP3R1 (red). Nuclei were counterstained with DAPI (blue); original magnification, 63x; scale bars, 10 μm; EC: endothelial cells (yellow arrows); VSMC: vascular smooth muscle cells (Cyan arrows). Individual data are shown with mean ± s.e.m. of three independent experiments; A.U.: arbitrary units. *: p<0.01 *vs* IP3R1$^{fl/fl}$; two-tailed Student's *t* test.

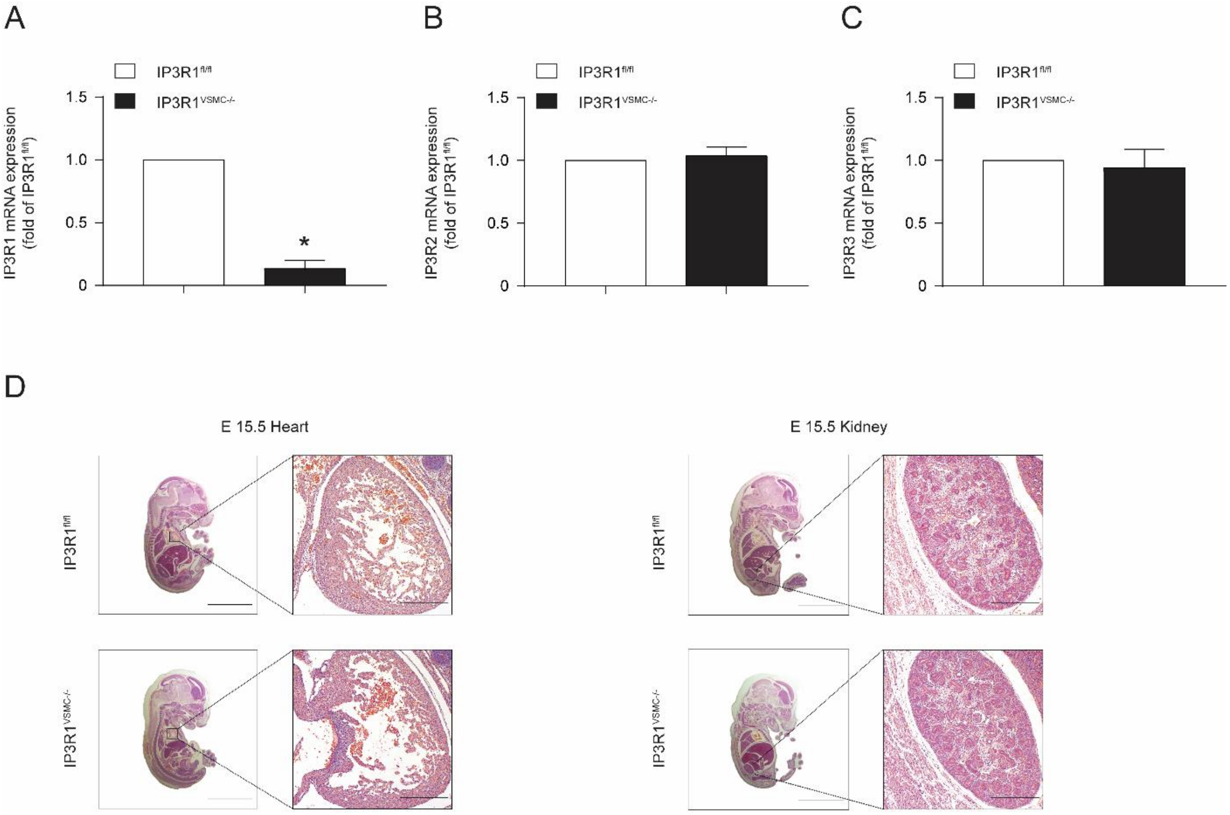

Figure S2. No upregulation of IP3R2 IP3R3 isoforms or abnormal organs development in IP3R1 deficient VSMC. IP3R1, IP3R2 and IP3R3 mRNA levels quantified by real-time RT-qPCR analysis of total RNA from isolated VSMC, using actin as internal standard; each bar represents mean ± s.e.m. of four independent experiments in each of which reactions were performed in triplicate using the pooled total RNAs from five mice/group; *: p<0.01 *vs* IP3R1$^{fl/fl}$; two-tailed Student's *t* test. **D)** Normal organ development in IP3R1$^{VSMC-/-}$ mice. Representative pictures of heart and kidney at embryonic day 15.5 are shown; no developmental abnormalities were observed in any of the major organs. Dimensional bar: 4 mm (in each inset: 200 μm).

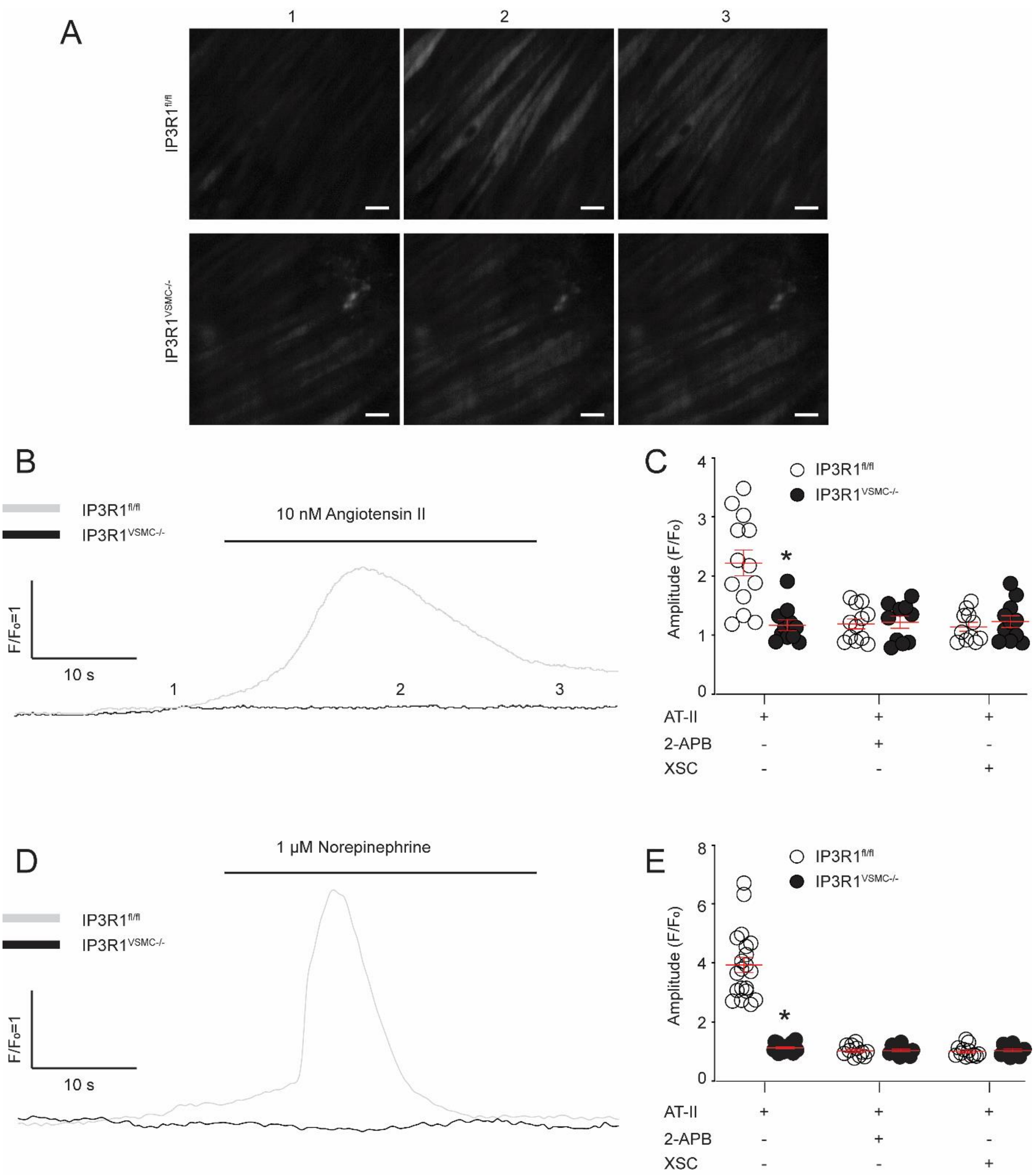

Figure S3. Effects of IP3R1 deletion on Ca^{2+} responses in VSMC.

A) Representative images of VSMC Ca^{2+} response to ATII (pictures labeled as 1, 2, and 3 correspond to the representative Ca^{2+} traces shown in panel **B,** measured with fluorescent Ca^{2+} indicator Fluo-4**;** F/F_0 is quantified in panel **C,** showing also the effects of 2-aminoethoxydiphenyl borate (2-APB) and xestospongin C (XSC)**. D)** Representative traces of VSMC Ca^{2+} responses to NE, quantified in panel **E**. Data are presented as individual values with means ± SEM. n = at least 30 cells from 6 different mice per group, **p* <0.05 *vs* IP3R1$^{fl/fl}$. two-tailed Student's *t* test. scale bars, 10 μm.

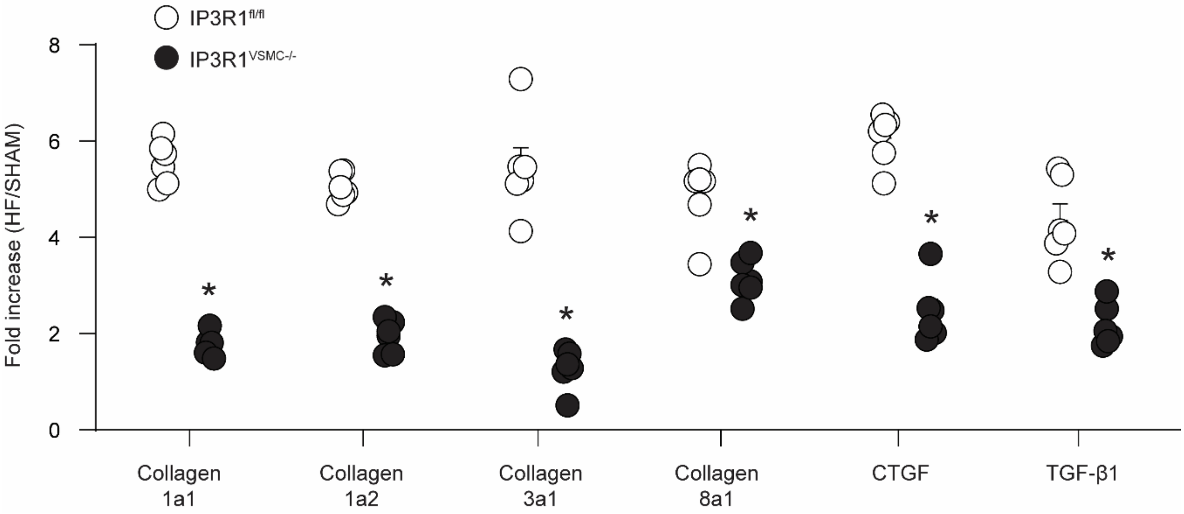

Figure S4. **Reduced mRNA levels of collagens, connective tissue growth factor (CTGF), and transforming growth factor β1 (TGF-β1)** assessed using real-time RT-qPCR analysis of total RNA from cardiac tissue, using actin as internal standard; Individual values are shown with mean ± s.e.m. of four independent experiments in each of which reactions were performed in triplicate using the pooled total RNAs from at least five mice/group; *:$p<0.05$ *vs* IP3R1$^{fl/fl}$. two-tailed Student's *t* test.

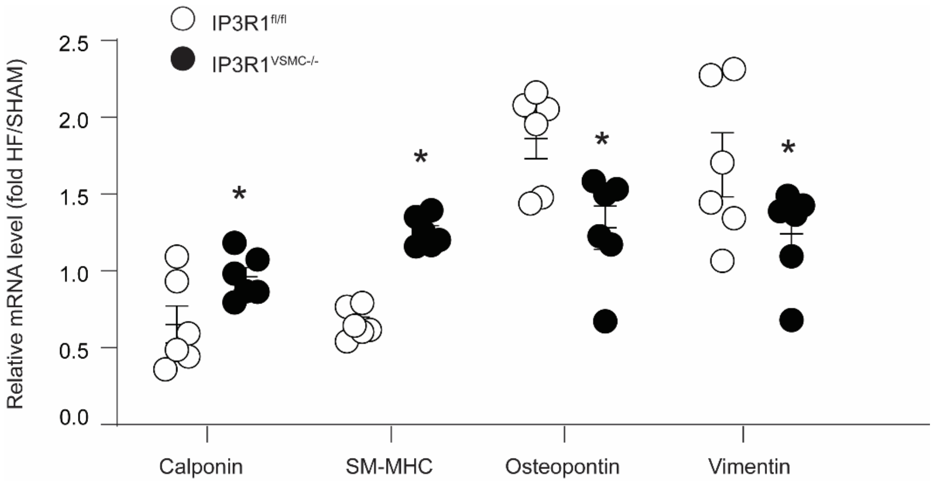

Figure S5. Upregulation of the quiescent (calponin, SM-MHC) and down regulation of the proliferative state (osteopontin, vimentin). calponin, SM-MHC, osteoponin and vimentin, in HF and SHAM conditions assessed using real-time RT-qPCR analysis of total RNA, using actin as internal standard; Individual values are shown with mean ± s.e.m. of four independent experiments in each of which reactions were performed in triplicate using the pooled total RNAs from at least five mice/group; *:$p<0.05$ *vs* IP3R1$^{fl/fl}$. two-tailed Student's *t* test.

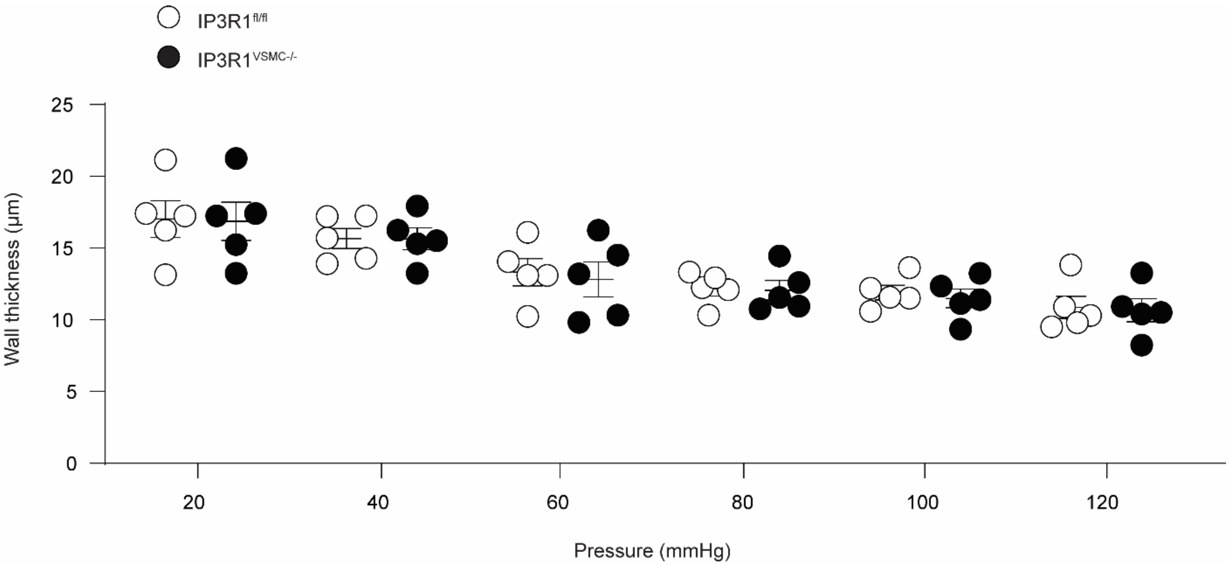

Figure S6: Measurement of wall thickness in the myography assays shown in Figure 4. The difference in the myogenic contractile response between IP3R$^{fl/fl}$ and IP3R1$^{VSMC-/-}$ mesenteric arteries (shown in Figure 4), cannot be attributed to modifications in wall thickness; n>5/group. Individual values are shown with mean± s.e.m. two-tailed Student's *t* test

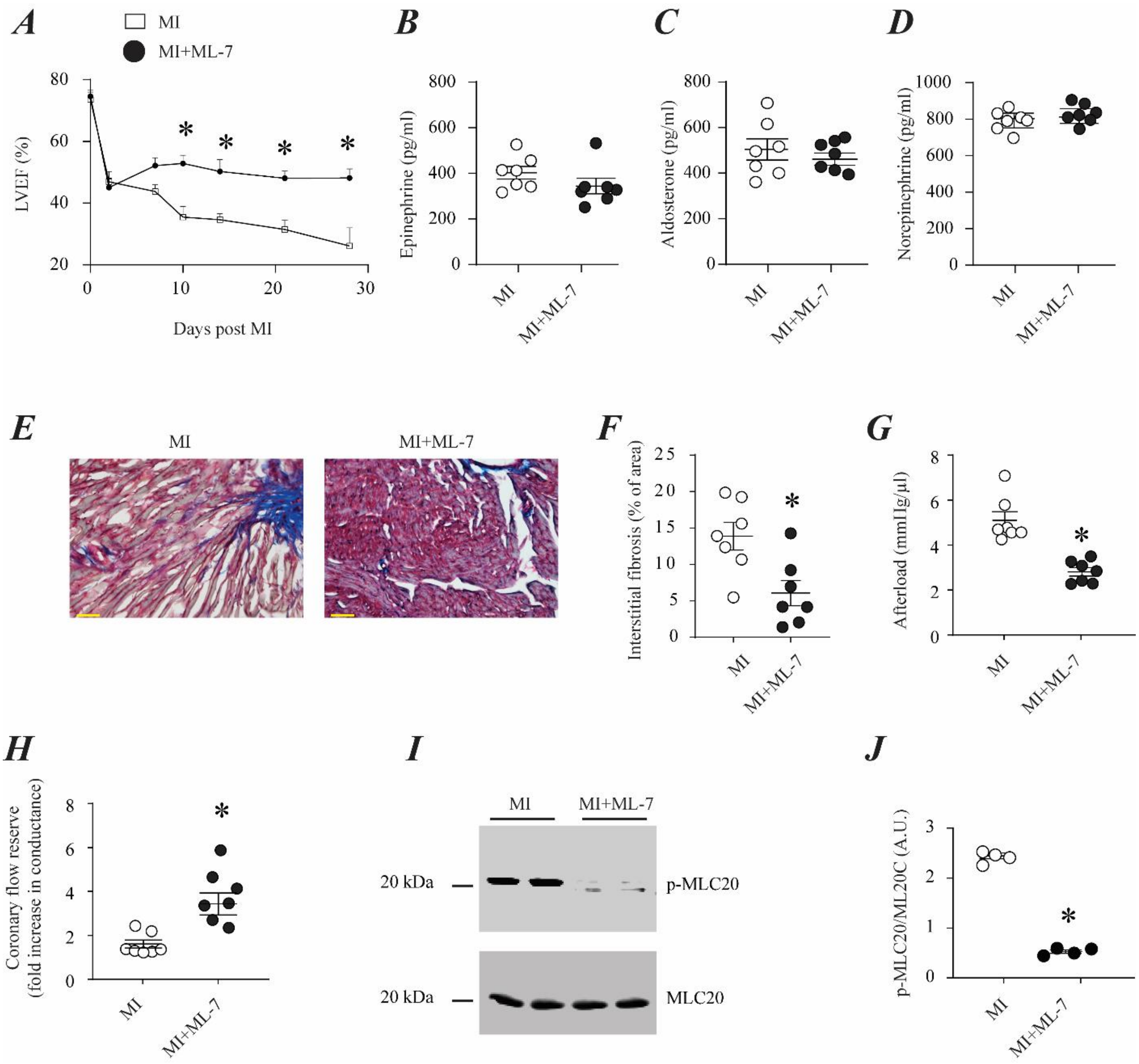

Figure S7: MLCK inhibitor (ML-7) reduces HF progression. A) HF mice treated with MLCK inhibitor ML-7 exhibit improved left ventricular ejection fraction (LVEF, evaluated by serial echocardiography). **B-D**) ML-7 has no effect on neurohormonal activation in HF assessed by measuring blood level of catecholamines and aldosterone; **E-F**) representative images and bar graph showing reduced interstitial cardiac fibrosis (Masson's trichrome staining, scale bar: 50µm) in MI mice treated with ML-7. **F**; **G**) Reduced cardiac afterload in ML-7 treated MI mice (ratio of end-systolic pressure and stroke volume); **H**) Improved coronary flow reserve determined in ML-7 treated MI mice; **I-J)** immunoblots of denuded mesenteric arteries ($\geq$7 mice per group) showing reduced MLC protein phosphorylation levels in WT MI mice treated with ML-7 quantified in **J.** Individual values are shown with means ± s.e.m.; A.U.: arbitrary units. *$p < 0.05$, WT control compared to post-MI mice. ANOVA repeated measures and two-tailed Student's *t* test

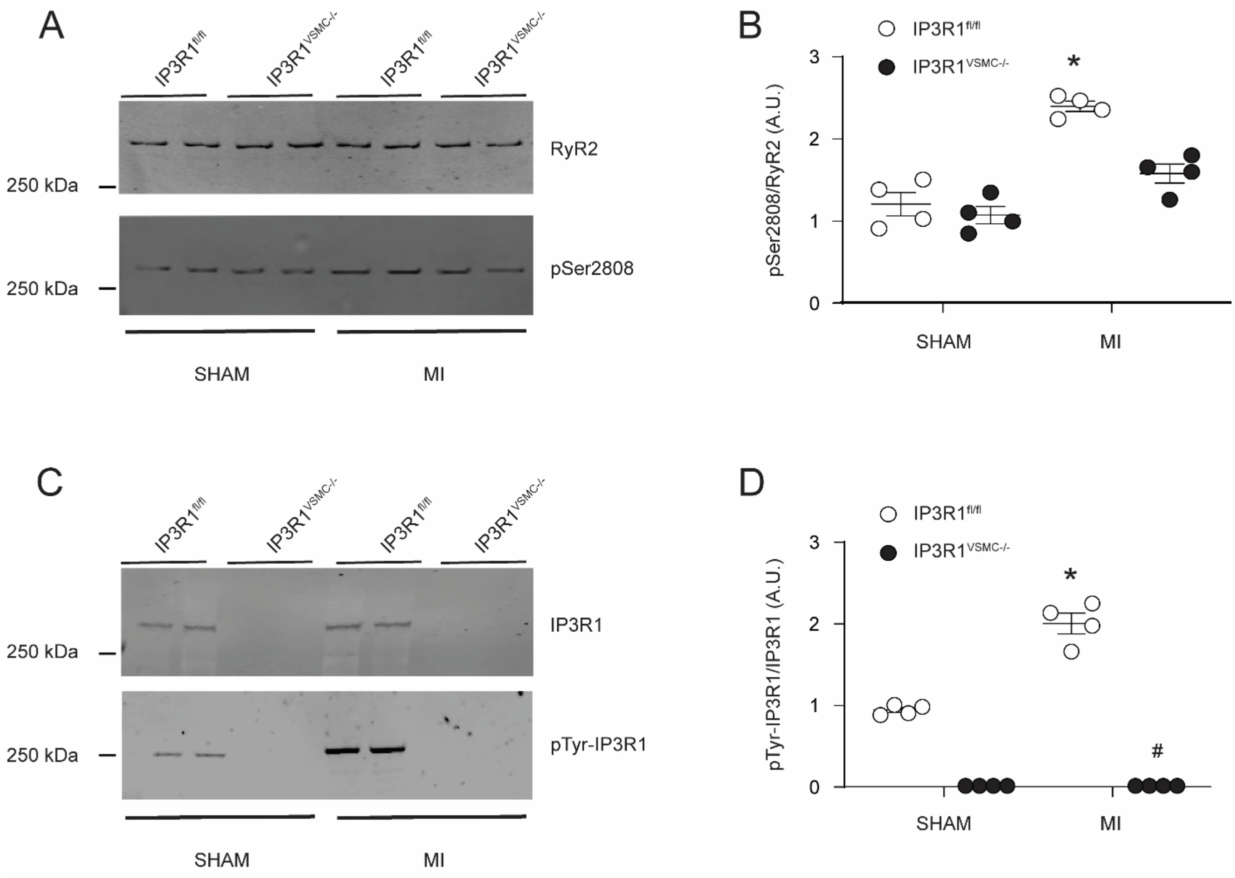

Figure S8. RyR2 and IP3R1 phosphorylation by PKA and tyrosine kinases in VSMC. A-B) phosphorylation of RyR2 by PKA on Serine^{2808} in denuded mesenteric arteries from IP3R1$^{fl/fl}$ and IP3R1$^{VSMC-/-}$ SHAM and MI. **C-D)** Phosphorylation of IP3R1 by tyrosine kinases in denuded mesenteric arteries from IP3R1$^{fl/fl}$ and IP3R1$^{VSMC-/-}$ SHAM and MI. Individual values are shown with mean± s.e.m.; A.U.: arbitrary units. *p<0.05 *vs* IP3R1$^{fl/fl}$ SHAM, #p<0.05 vs. IP3R1$^{fl/fl}$ MI. two-tailed Student's *t* test.

	Age (year)	Sex	Lane number in Figure 1	LVEF (%)	Neurological disorders
**1641 (control)**	68	Male	**1**	45	None
**1627 (control)**	66	Male	**2**	55	None
**1576 (control)**	32	Male	**3**	58	None
**H54 (Control)**	58	Male	**4**	60	None
**Z46 (HF)**	67	Male	**5**	30-45	None
**H40 (HF)**	64	Male	**6**	30	None
**Z39(HF)**	32	Male	**7**	20-25	None
**Z111 (HF)**	66	Male	**8**	20	None
**1080 (HF)**	47	Female	**9**	10-15	None

Table S1: Characteristics of HF patients and controls biopsies. Z46, Z39, and 1080 had multiple values from different echocardiographic examinations; therefore, we are showing the min-max range of their left ventricular ejection fraction (LVEF). The lane number corresponds to the position of the sample in the gels in Figure 1.

Parameter	SHAM		MI	
	IP3R1$^{fl/fl}$	IP3R1$^{VSMC-/-}$	IP3R1$^{fl/fl}$	IP3R1$^{VSMC-/-}$
BW, g	25.2±1.3	24.8±1.5	25.1±1.5	25.1±1.6
HR, bpm	504±64	512±68	492±72	496±84
HW/BW, mg/g	4.81±0.71	5.01±0.85	8.03±0.83$^{\#}$	7.7±0.8$^{*,\#}$
LW/BW, mg/g	5.5±0.65	5.64±0.84	7.25±0.75$^{\#}$	6.85±0.7$^{*,\#}$
LVEF, %	79.6±3.6	78.9±4.3	36.8±3.4$^{\#}$	46.9±3.1$^{*,\#}$
LVFS, %	49.8±1.6	49.5±1.7	18.9±1.7$^{\#}$	23.4±1.6$^{*,\#}$
dP/dt_{max}	7720±582	7742±594	5864±448$^{\#}$	6712±422$^{*,\#}$
LVSBP, mmHg	110.8±2.8	111.3±3.5	107.1±3.8	106.7±3.2
Serum TnI, ng/ml	-	-	61.2±13.3	62.4±13.8
Infarct size, % of LV	-	-	42.1±5.2	42.8±5.6

Table S2. Characteristics of SHAM and MI mice.
BW: body weight; dP/dt_{max}: maximum derivative of change in pressure rise over time; HW: heart weight; HR: heart rate; LV: left ventricle; LVEF: left ventricle ejection fraction; LVFS: left ventricle fractional shortening; LVSBP: left ventricle systolic blood pressure; LW: lung weight; TnI: Troponin I; n>8 mice/group; *: $p<0.05$ vs IP3R1$^{fl/fl}$, $^{\#}$: $p<0.05$ vs SHAM, ANOVA, Tukey-Kramer *post hoc* test.

	MI	MI+ML-7
BW, g	30.4±1.9	30.9±1.1
HR, bpm	566±34	562±33
LVSBP, mmHg	106±15	110±15
LVDBP, mmHg	81±12	79±14
HW/BW, mg/g	7.1±1.9	5.8±0.6*
LW/BW, mg/g	4.4±0.3	4.5±0.2
LVEF, %	26±5.8	48±3.15*
LVFS, %	12±3.2	24±1.9*
dP/dt_{max}	6213±235	7130±346*
Infarct size, % of LV	43.4±6.0	44.3±4.9

Table S3. Characteristics of MI and MI+ML-7 mice.
BW: body weight; dP/dt_{max}: maximum derivative of change in pressure rise over time; HW: heart weight; HR: heart rate; LV: left ventricle; LVEF: left ventricle ejection fraction; LVFS: left ventricle fractional shortening; LVSBP: left ventricle systolic blood pressure; LW: lung weight; TnI: Troponin I; n = >7 mice/group; *: $p<0.05$ vs HF untreated.

Gene	Forward 5'-3'	Reverse 5'-3'
IP3R1	TGGTCCAGCACTTTGTTCAC	TCTGCCTTGACAATCGTCTG
IP3R2	AGACTCTCAGCTCGCTCTGG	GGCCACGACATCCTGTAACT
IP3R3	ACATCCTGGCTGAAGACACC	AAAGGTCTCCACCTCCGTCT
Calponin	GCACATTTTAACCGAGGTCC	ATGGACACAAGTGCTAAGCAGTCT
CTGF	AAGCTGACCTGGAGGAAAACA	TGCAGCCAGAAAGCTCAAAC
Col1a1	TTCAGGGAATGCCTGGTGAA	ACCTTTGGGACCAGCATCA
Col1a2	GAAAAGGGTCCCTCTGGAGAA	AATACCGGGAGCACCAAGAA
Col3a1	TGCTGGAAAGAATGGGGAGAC	GGTCCAGAATCTCCCTTGTCAC
Col8a1	CCAGCCCCAGTGGTATTACA	ACAGTATTCCCAGCAGCTGTA
Osteopontin	CAGGACAACAACGGAAAGGG	CCTGGCTCTCTTTGGAATGC
TGF-β1	GCTGCGCTTGCAGAGATTAA	GTAACGCCAGGAATTGTTGCTA
SM-MHC	TGGACACCATGTCAGGGAAA	AGTCGGCATCGTTTATGGTC
Vimentin	AGGAAATGGCTCGTCACCTTCGTGAATA	GGAGTGTCGGTTGTTAAGAACTAGAGCT
Actin	CTCTTCCAGCCTTCCTTCCT	AGCACTGTGTTGGCGTACAG

Table S4. Primer sequences. Col: collagen isoforms; CTGF: connective tissue growth factor; SM-MHC: smooth muscle myosin heavy chain; TGF-β1: transforming growth factor β1.
